# Idiopathic intracranial hypertension presenting with obsessive thoughts: a rare neuropsychiatric manifestation “case report”

**DOI:** 10.1186/s12883-026-04895-5

**Published:** 2026-06-01

**Authors:** Mennatallah Mohamed Rehab, Amr S. Zaki, Mohamed Gamal Farwiez, Ramez Reda Moustafa

**Affiliations:** Department of Neurology, Faculty of Medicine, Ain Shams University, Cairo, Egypt

**Keywords:** Idiopathic intracranial hypertension, Obsessive compulsive symptoms, Venous sinus stenosis, Venous sinus stenting, Neuropsychiatric manifestation, Case report

## Abstract

**Background:**

Idiopathic intracranial hypertension (IIH) classically presents with headache, papilledema, and visual disturbances. Psychiatric manifestations are rare and may obscure a timely diagnosis.

**Case presentation:**

A 23-year-old obese female with no prior psychiatric history presented with new-onset intrusive thoughts and compulsive finger-tapping rituals, leading to a diagnosis of obsessive–compulsive disorder (OCD). Despite treatment with high-dose SSRIs, antipsychotic augmentation, and cognitive-behavioural therapy for six months, she showed no improvement. She concurrently reported a refractory bitemporal headache, tinnitus, and transient visual obscurations. Examination revealed bilateral papilledema. MRI/MRV showed features of raised intracranial pressure (ICP) associated with right transverse sinus stenosis. Lumbar puncture (LP) confirmed an elevated opening pressure of 30 cm H₂O. Despite treatment with acetazolamide (2 g/day), she remained symptomatic. Venous sinus stenting was subsequently performed, restoing sinus caliber and reducing the pressure gradient. Within three months, her headache improved markedly, and her papilledema resolved. Remarkably, her obsessive thoughts and compulsive acts completely disappeared without psychiatric medication, with her Yale-Brown Obsessive–Compulsive Scale (Y-BOCS) score dropping from 20 to 5.

**Conclusion:**

This case illustrates IIH presenting with predominant psychiatric manifestations mimicking primary OCD. The dramatic resolution of compulsive symptoms following intracranial pressure normalization suggests a possible association between IIH and obsessive thoughts. Clinicians should maintain a high index of suspicion for IIH in atypical psychiatric presentations accompanied by headache, tinnitus, or visual symptoms; in such cases, routine fundoscopy is essential.

## Background

Idiopathic intracranial hypertension (IIH) is characterized by raised intracranial pressure (ICP) of unknown cause, typically affecting obese young women and presenting with headache, papilledema, and visual disturbances. While cognitive complaints and mood disturbances have occasionally been described, primary psychiatric presentations are exceedingly rare and often lead to delayed diagnosis. Obsessive–compulsive disorder (OCD) is a neuropsychiatric condition involving intrusive thoughts and compulsive behaviours, with established neurobiological underpinnings in frontostriatal circuits. The emergence of de novo obsessive–compulsive symptoms in the context of IIH has not been well documented. Recognition of such atypical manifestations is crucial for the timely identification and management of secondary causes. We report the case of a young woman initially diagnosed with primary OCD who was later found to have IIH with venous sinus stenosis. Remarkably, her compulsions resolved completely following venous sinus stenting and normalization of intracranial pressure. This case highlights the potential neuropsychiatric dimension of IIH and underscores the importance of considering organic causes in atypical psychiatric presentations.

## Case presentation

A 23-year-old obese woman with a body mass index (BMI) of 30 kg/m^2^ and no prior psychiatric history presented with a three-month history of new-onset intrusive thoughts. The patient's obsessive thoughts centered on fears of contamination and causing harm to others through carelessness. She experienced recurrent intrusive thoughts that she had left doors unlocked or appliances on, despite knowing logically that she had checked them. To neutralize these thoughts, she developed elaborate finger-tapping rituals: she would tap each finger to her thumb in sequence, repeating the sequence a specific number of times (always multiples of four) until she felt a sense of 'completeness.' The rituals were time-consuming, occupying 2–3 h daily, and were associated with significant distress and functional impairment. She recognized the thoughts as irrational (good insight) but felt unable to resist the compulsions. Notably, the content of her obsessions was typical for OCD, but the onset was unusually acute (over 2–3 weeks) and occurred without prior personal or family history—features that should have prompted consideration of an organic etiology. She sought medical advice at a psychiatric institute, where she was diagnosed with obsessive–compulsive disorder (OCD). Her initial assessment using the Yale-Brown Obsessive–Compulsive Scale (Y-BOCS) [1] yielded a score of 22 (moderate OCD), and her Taylor Manifest Anxiety Scale (TMAS) [2] score was 33.

She received systematic psychopharmacological treatment under psychiatric supervision. Initial treatment with escitalopram was initiated at 10 mg/day and titrated over 8 weeks to 40 mg/day (maximum recommended dose), maintained for 12 weeks with no clinically significant improvement (Y-BOCS remained 20–22). Due to non-response, she was cross-titrated to fluvoxamine, reaching 300 mg/day (maximum dose) over 6 weeks, maintained for an additional 8 weeks. When response remained inadequate, aripiprazole was added at 5 mg/day, titrated to 15 mg/day over 4 weeks, and continued for 8 weeks—again without meaningful improvement. Concurrently, she completed 20 weekly sessions of exposure and response prevention (ERP) therapy with a certified cognitive-behavioral therapist, focusing on exposure to contamination triggers and prevention of tapping rituals.

During her continuous psychiatric follow-up, she consistently reported a headache refractory to analgesics. The pain was bitemporal, dull-aching, and severe (10/10 on the numerical rating scale). Her Headache Impact Test (HIT-6) score was 72 (severe). The headache worsened with Valsalva maneuvers, was more prominent in the morning, and was associated with unilateral tinnitus and transient visual obscurations.

**Fig. 1:**
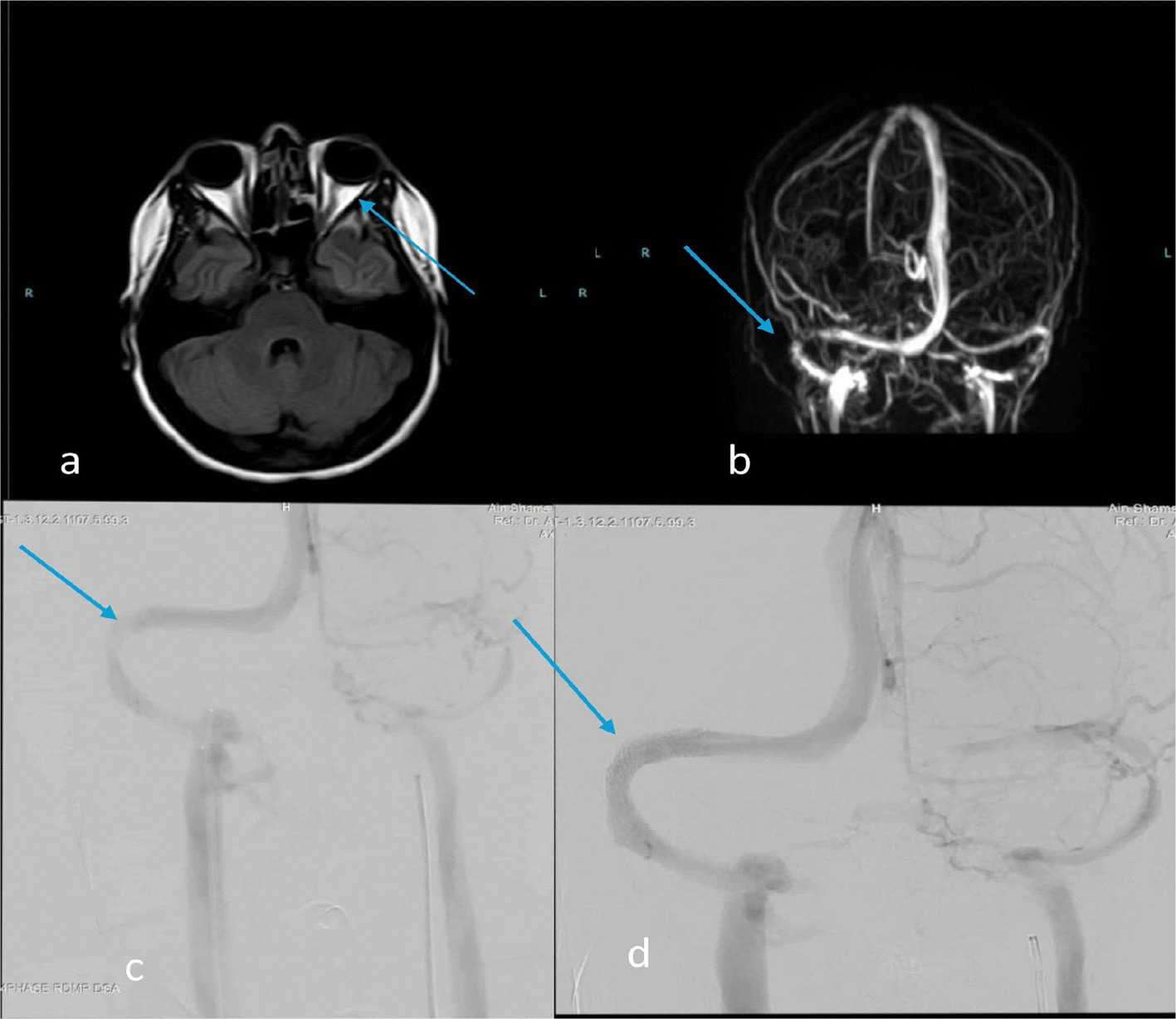
**a** axial T1 weighted brain MRI showing distention of optic nerve sheath complex. **b** MR venography demonstrating focal stenosis of right transverse sigmoid sinus junction. **c** Digital subtraction venography confirming significant stenosis. **d** Post-stenting DSV image showing restoration of normal venous outflow across the previously stenotic segment

On examination, bilateral papilledema was noted. Magnetic resonance imaging (MRI) of the brain and magnetic resonance venography (MRV) demonstrated normal brain parenchyma with a partially empty sella turcica, tortuous optic nerves (Fig. 1A) with increased perioptic CSF signal, and focal stenosis of the right transverse sinus (Fig. 1B); venous sinus thrombosis was ruled out. Lumbar puncture (LP) confirmed an elevated opening pressure of 30 cm H₂O with normal cerebrospinal fluid (CSF) composition.

A diagnosis of idiopathic intracranial hypertension (IIH) was made according to the International Classification of Headache Disorders, 3rd edition (ICHD-3). She showed marked improvement in her OCD symptoms following her lumbar puncture; however, symptoms recurred weeks later. She was prescribed acetazolamide (2,000 mg/day in four divided doses). Despite medication compliance, her headache and fundoscopic examination showed no improvement. A follow-up lumbar puncture three months later revealed a persistent opening pressure of 29 cm H₂O. Digital subtraction angiography (DSA) demonstrated a hypoplastic left lateral sinus and significant stenosis of the right transverse sinus, with a pressure gradient of 12 mmHg. The patient underwent right transverse sinus stenting, final angiography was performed to confirm the patency of the vein of Labbé, stent apposition, and the absence of residual stenosis. The pressure gradient was remeasured post-deployment. Fig. 1c,d).

She showed a remarkable response following the stenting procedure, particularly regarding her compulsions. During regular follow-ups, her clinical status significantly improved. At the three-month follow-up, her headache intensity decreased to 3/10 on the numerical rating scale, and her HIT-6 score improved to 46. Fundus examination showed a complete resolution of papilledema. Notably, her obsessions and compulsive acts completely ceased without the need for psychiatric medications, with her Y-BOCS score decreasing to 5.

## Discussion

We report a unique case of a 23-year-old obese woman with new-onset obsessive–compulsive disorder (OCD) as the presenting manifestation of idiopathic intracranial hypertension (IIH), which resolved completely following normalization of ICP. The lack of response to conventional psychiatric treatment suggests that the obsessive thoughts were secondary to raised ICP rather than a primary psychiatric disorder. This case highlights a novel and previously unreported association between IIH and OCD, and provides important insights into the pathophysiology of both conditions. Our case is unique in three respects: (1) OCD was the presenting and predominant clinical manifestation, leading to an initial psychiatric diagnosis; (2) the symptoms were severe and completely refractory to six months of optimized psychiatric treatment; and (3) the dramatic and sustained resolution following ICP normalization provides the strongest evidence to date for a causal relationship between IIH and obsessive–compulsive phenomena.The relationship between IIH and psychiatric comorbidities is well-established. Mollan et al., in a large population-based matched cohort study, demonstrated that women with IIH have a significantly higher hazard of developing depression and anxiety compared to population controls (aHR 1.38 and 1.40, respectively), with a burden comparable to that of women with migraine [3]. Similarly, Donaldson et al. found that symptoms of anxiety and depression are highly prevalent in IIH patients, with over half reporting at least mild symptoms, though they noted that these were often under-reported and correlated more strongly with younger age and higher body mass index (BMI) than with the IIH diagnosis itself [4].

Our patient, with a BMI of 30 kg/m^2^ and significant functional impairment, fits this demographic profile. However, while these studies focused on depression and generalized anxiety, our case extends the psychiatric spectrum associated with IIH to include a full-blown, treatment-refractory OCD. The complete and sustained resolution of the patient's OCD symptoms following cerebrospinal fluid (CSF) diversion—first temporarily after lumbar puncture and then permanently after venous sinus stenting—provides s evidence for a causal, rather than merely coincidental, relationship. This dramatic response suggests that the elevated intracranial pressure (ICP) may have contributed to driving the obsessive–compulsive phenomena.

Several alternative explanations for the patient's obsessive–compulsive symptoms warrant consideration. First, the psychological stress of chronic headache, visual disturbances, and functional impairment could have precipitated obsessive thoughts and compulsive behaviors as maladaptive coping mechanisms. However, the persistence of symptoms despite six months of targeted CBT and the absence of prior psychiatric history argues against this as the primary explanation. Second, medication-induced behavioral effects must be considered; although SSRIs can rarely induce or exacerbate anxiety, our patient's symptoms predated treatment and did not improve with medication changes or discontinuation. Third, sleep disruption from nocturnal headaches and tinnitus could have contributed to executive dysfunction and repetitive behaviors, though again, the symptom profile was classic for OCD rather than general cognitive impairment. Fourth, it is possible that IIH and OCD co-occurred by chance in a young woman with obesity, a risk factor for both conditions. While we cannot definitively exclude this possibility, the temporal relationship between ICP normalization and symptom resolution—with temporary improvement after lumbar puncture and complete, sustained remission after stenting—strongly supports a pathophysiological link rather than mere coincidence.

The differential diagnosis of new-onset obsessive–compulsive symptoms in a young adult is broad and includes primary psychiatric disorders, secondary OCD from neurological conditions, and medication-induced phenomena. Primary OCD was initially diagnosed by a psychiatrist based on DSM-5 criteria, including the presence of intrusive thoughts, compulsive rituals, insight, and significant distress. However, several features should have raised suspicion for a secondary cause: (1) absence of personal or family history of OCD; (2) complete refractoriness to two different SSRIs at maximum doses, antipsychotic augmentation, and 20 sessions of ERP—a treatment combination that typically produces at least partial response in primary OCD; (3) concurrent atypical headache with features of raised ICP; (4) transient improvement after lumbar puncture. Neurological causes of obsessive–compulsive symptoms include structural lesions (e.g., frontostriatal tumors, basal ganglia calcification), autoimmune encephalitis (e.g., anti-NMDA receptor encephalitis, Sydenham chorea), neurodegenerative disorders (e.g., Huntington disease), and metabolic/toxic conditions. These were excluded by normal brain MRI, absence of movement disorder, and normal CSF composition.

The decision to proceed to venous sinus stenting was guided by: (1) persistent severe symptoms despite maximum medical therapy (acetazolamide 2 g/day for 3 months); (2) evidence of significant venous sinus stenosis with a trans-stenotic pressure gradient of 12 mmHg on DSA; (3) recurrent elevation of ICP on follow-up LP (29 cm H₂O); and (4) the patient's preference for a definitive intervention after discussing options including CSF shunting.

The pathophysiological mechanism linking raised ICP to OCD symptoms can be hypothesized by integrating findings from the IIH and OCD neuroimaging literature. Sarica et al., using diffusion tensor imaging (DTI), demonstrated significant microstructural alterations in the periventricular white matter of patients with IIH, specifically noting decreased mean, axial, and radial diffusivity in the corpus callosum and corona radiata [5]. These changes, which correlated negatively with CSF pressure levels, were interpreted as evidence of tissue compaction from mechanical compression against the ventricular walls [5]. Importantly, these white matter tracts are critical for interhemispheric communication and fronto-striatal connectivity. The prevailing model of OCD pathophysiology, as detailed by Milad and Rauch, emphasizes dysfunction within cortico-striato-thalamo-cortical (CSTC) loops, involving the orbitofrontal cortex (OFC), anterior cingulate cortex (ACC), and striatum [4]. They propose that hyperactivity in the lateral OFC may mediate obsessions, while deficient function in the medial OFC and ventromedial prefrontal cortex (vmPFC) impairs fear extinction and behavioral inhibition [6]. We hypothesize that in our patient, the mechanical compression from elevated ICP, as evidenced by the DTI changes described by Sarica et al. [5], disrupted the integrity of these critical fronto-striatal and interhemispheric circuits. This disruption could have precipitated the new-onset intrusive thoughts and compulsive rituals by altering the normal processing of behavioral inhibition and error monitoring, functions typically subserved by the ACC and OFC [6]. The normalization of ICP through stenting would then relieve this mechanical distortion, allowing the affected neural circuits to return to normal function and leading to the observed resolution of OCD symptoms. However, without pre- and post-stenting functional neuroimaging in our patient, this remains speculative, and alternative explanations cannot be excluded.

The decision to pursue venous sinus stenting was guided by the patient's persistent symptoms and evidence of a significant trans-stenotic pressure gradient. The 2022 IIH consensus guidelines acknowledge that while CSF diversion (e.g., ventriculoperitoneal shunting) is the preferred surgical procedure for visual loss in the UK, venous sinus stenting may be useful for highly selected patients with venous sinus stenosis and an elevated pressure gradient [7]. Our patient met these criteria, with digital subtraction angiography (DSA) revealing a pressure gradient of 12 mmHg. Zhang et al. have recently validated the use of DSA-derived hemodynamic features to predict a trans-stenotic gradient of ≥ 8 mmHg, supporting the hemodynamic significance of the stenosis in our patient [6]. The prospective trial by Dinkin and Patsalides further corroborates the efficacy and safety of this approach, demonstrating that stenting leads to significant improvements in headache, papilledema, visual fields, and a mean reduction in opening pressure of 20 cmH₂O [8]. Our patient's post-stenting outcomes—resolution of headache, papilledema, and normalization of the Y-BOCS score—align perfectly with these reported benefits. The role of stress and its physiological mediators in headache and psychiatric disorders is also relevant. Sic et al. review how chronic stress dysregulates the hypothalamic–pituitary–adrenal (HPA) axis and autonomic nervous system, leading to neuroinflammation and central sensitization [9]. While this model is typically applied to primary headache disorders, it is plausible that the physical stress of chronically elevated ICP on the brain parenchyma could similarly contribute to a state of neuronal hyperexcitability and dysfunction within the anxiety and fear circuits described by Milad and Rauch [4], thereby lowering the threshold for the development of OCD. As Donaldson et al. highlight, psychiatric symptoms are frequently under-reported, and a high index of suspicion is necessary [4]. This case has several limitations. As a single case report, the findings cannot be generalized.

Furthermore, we propose the following hypothetical mechanism linking raised ICP to OCD symptoms, which requires validation in future studies. Drawing on DTI findings from Sarica et al. [5] demonstrating periventricular white matter microstructural changes in IIH, we speculate that elevated ICP may mechanically compress critical fronto-striatal and interhemispheric white matter tracts. These tracts are known to be involved in OCD pathophysiology, particularly in circuits mediating behavioral inhibition and error monitoring [6]. If this hypothesis is correct, normalization of ICP through stenting would relieve this mechanical distortion, allowing affected neural circuits to return to normal function. This framework could explain the observed temporal relationship between ICP normalization and symptom resolution. However, without pre- and post-stenting functional neuroimaging in our patient, this remains speculative, and alternative explanations cannot be excluded. Future research should explore the prevalence of OCD in larger IIH cohorts and investigate the potential for IIH to masquerade as a primary psychiatric disorder. In conclusion, this case demonstrates that new-onset, treatment-refractory OCD can be the predominant clinical manifestation of IIH. The complete resolution of psychiatric symptoms following venous sinus stenting underscores the critical importance of considering IIH in the differential diagnosis of atypical psychiatric presentations, particularly in young, obese women. It also reinforces the role of venous sinus stenting as an effective treatment for carefully selected patients with IIH and a significant trans-stenotic pressure gradient.

Clinical implications and take-home points for clinicians:

- 1. When to suspect a neurological cause in psychiatric presentations: Consider secondary causes in patients with: (a) new-onset psychiatric symptoms after age 30; (b) absence of personal or family psychiatric history; (c) treatment-refractory course despite adequate trials; (d) atypical symptom features; (e) concurrent neurological symptoms (headache, visual disturbances, tinnitus).
- 2. Red flags for IIH in psychiatric settings: In young, overweight/obese patients presenting with anxiety, depression, or (rarely) obsessive–compulsive symptoms, ask specifically about: (a) headache (particularly morning exacerbation, Valsalva-induced worsening); (b) transient visual obscurations; (c) pulsatile tinnitus; (d) diplopia.
- 3. Role of fundoscopy: Fundoscopic examination is a simple, non-invasive, and inexpensive bedside tool that can detect papilledema. We advocate for routine fundoscopy in all patients with atypical psychiatric presentations, particularly those with any of the above red flags. A normal fundus does not exclude IIH (papilledema may be absent in early or mild cases), but an abnormal finding should prompt urgent neurological referral.
- 4. Diagnostic approach: Patients with suspected IIH should undergo brain imaging (MRI/MRV) to rule out venous sinus thrombosis and identify features of raised ICP (empty sella, optic nerve tortuosity), followed by lumbar puncture with opening pressure measurement if imaging is non-controversial.

## Data Availability

All data relevant to this case report are included in the manuscript. Additional clinical data are not publicly available to protect patient privacy but may be available from the corresponding author upon reasonable request and with appropriate ethical approvals.

## Abbreviations

IIH: Idiopathic intracranial hypertension
OCD: Obsessive–compulsive disorder
SSRIs: Selective serotonin reuptake inhibitors
MRI: Magnetic resonance imaging
MRV: Magnetic resonance venography
BMI: Body mass index
Y-BOCS: Yale brown obsessive–compulsive scale
TMAS: Taylor Manifest anxiety scale (TMAS)
HIT-6: Headache impact test
CSF: Cerebrospinal fluid
LP: Lumbar puncture
ICHD-3: International classification of headache disorder, 3rd edition (ICHD-3)
DSA: Digital subtraction angiography
ICP: Intracranial pressure
